# Artificial Intelligence agents for biological research: a survey

**DOI:** 10.1093/bib/bbag075

**Published:** 2026-02-26

**Authors:** Cong Qi, Wenbo Wang, Siqi Jiang, Qin Liu, Xun Song, Hanzhang Fang, Zhi Wei

**Affiliations:** Department of Computer Science, New Jersey Institute of Technology, 323 Dr Martin Luther King Jr Blvd, Newark, NJ 07102, United States; Department of Computer Science, Hamilton College, 198 College Hill Road, Clinton, NY 13323, United States; Department of Computer Science, New Jersey Institute of Technology, 323 Dr Martin Luther King Jr Blvd, Newark, NJ 07102, United States; Martin Tuchman School of Management, New Jersey Institute of Technology, 323 Dr Martin Luther King Jr Blvd, Newark, NJ 07102, United States; Department of Computer Science, New Jersey Institute of Technology, 323 Dr Martin Luther King Jr Blvd, Newark, NJ 07102, United States; Department of Computer Science, New Jersey Institute of Technology, 323 Dr Martin Luther King Jr Blvd, Newark, NJ 07102, United States; Department of Computer Science, New Jersey Institute of Technology, 323 Dr Martin Luther King Jr Blvd, Newark, NJ 07102, United States

**Keywords:** computational biology, generative AI, bioinformatics

## Abstract

The rapid growth of biological data and experimental complexity has motivated increasing interest in artificial intelligence (AI) systems that extend beyond static prediction toward autonomous reasoning and action. While recent computational models achieve strong predictive performance, they largely operate as passive tools within human-driven research workflows. In contrast, AI agents integrate reasoning, planning, tool invocation, and feedback-driven refinement, enabling more adaptive and interactive forms of biological analysis. This survey provides a systematic synthesis of recent progress in biological AI agents by reviewing over 100 representative studies across clinical analytics, molecular and drug design, multi-omics analysis, and knowledge discovery. We introduce a unified 5D taxonomy that organizes existing work along task domains, system architectures, interaction modes, evaluation strategies, and resource integration. Building on this framework, we analyze common design patterns, highlight emerging capabilities enabled by agentic paradigms, and identify key open challenges, including reliability, privacy, scalability, and standardized evaluation. Collectively, this survey clarifies the conceptual and methodological landscape of biological AI agents and outlines directions toward more robust, transparent, and collaborative agent-based systems for biological research. To serve as a living resource for the community, we curated a GitHub repository that includes resources and benchmark summaries, available at https://github.com/MineSelf2016/biological_agents_survey.

## Introduction

Biological research is experiencing an unprecedented surge in both data volume and experimental complexity. From large-scale clinical text mining to single-cell transcriptomics (scRNA-seq), multi-omics integration, and molecular drug design, the central challenge has shifted beyond merely improving predictive accuracy to involving models actively contributing to scientific discovery [1–5]. While excelling at representational and predictive learning, current computational approaches typically follow a one-shot inference pattern: the model receives input, produces output, and the process terminates. Such a static prediction mechanism fails to meet the modern demands of biology for dynamic reasoning, process monitoring, and closed-loop validation.

In recent years, the widespread adoption of large language models (LLMs) has shifted researchers’ attention from prediction to reasoning. Powered by language models as their reasoning engines, artificial intelligence (AI) agents actively integrate external knowledge bases, analytical tools, and feedback signals to form an adaptive “perception–planning–action–reflection” loop [6, 7]. Within this loop, models evolve from passive predictors that only respond to inputs into autonomous participants capable of ongoing self-adjustment. Tool-augmented and retrieval-augmented frameworks, such as AutoGPT [8] and ReAct [9], have validated this “decisionverificationreplanning” paradigm in various domains, including chemistry, genetic engineering, and clinical medicine. In biological research, various agent-based systems demonstrate the potential of this new paradigm. For example, GeneAgent [4] enhances reproducibility by conducting self-verification against gene set databases; CellAgent [10] leverages multi-agent collaboration to facilitate end-to-end single-cell data analysis; while SpatialAgent [11] extends this agent-based architecture to spatial transcriptomics. Taken together, these developments reflect a shift from solely relying on stronger models to adopting cognitively organized research systems that act as collaborative partners [12, 13].

Despite its clear potential, the development of biological AI agents is still in its nascent stage, with relatively limited and fragmented studies. Meanwhile, existing agents are typically designed for single tasks or specific data modalities and are deployed in various contexts, such as clinical decision support, molecular design, and single-cell analysis. This leads to substantial heterogeneity in the design and application of the systems. Such fragmentation and heterogeneity highlight the urgent need for a systematic review to assist researchers in identifying research gaps and promising directions.

Therefore, this paper adopts a structured literature review to synthesize the existing studies on biological AI agents. We searched mainstream academic databases, including arXiv, PubMed, bioRxiv, and Google Scholar, for publications from 1 January 2023 to 30 September 2025. The search strategy encompassed the two main domains of AI and biology, identifying interdisciplinary research through various combinations of keyword phrases. After cross-searching and manual screening, 115 representative studies remained for analysis. Studies were included if they explicitly demonstrated agentic characteristics such as multi-step reasoning, autonomous task planning, persistent state or memory management, and closed-loop interaction with biological tools, databases, or experimental environments. In contrast, pure foundation models, single-turn retrieval-augmented generation (RAG)systems, or prompt-based pipelines without autonomous decision-making were excluded from the scope of this survey. We developed 5D of biological AI agent research: *task domains, architectural paradigms, evaluation strategies, interaction modes, and resource integration*, which serve as both a systematic synthesis of current research and a forward-looking analytical lens. Furthermore, we identify a set of challenges that must be addressed for biological AI agents to mature, such as data privacy, reliability, and evaluation. Finally, this survey points toward the development of biologically grounded, multi-agent, lightweight, and standardized AI systems that function as collaborative scientific partners, supporting robust, transparent, and widely accessible computational discovery.

Several recent surveys have reviewed the development of AI agents from a general perspective, focusing on architectural paradigms, reasoning and planning mechanisms, or multi-agent communication protocols [14–16]. These works provide valuable overviews of agent design and system capabilities, but are largely domain-agnostic and typically treat biological applications as illustrative case studies rather than as a primary focus. In addition, some recent works in the biomedical domain adopt a perspective-style treatment, emphasizing high-level visions of “AI scientists,” agent autonomy, and long-term research roadmaps, rather than providing a systematic survey of implemented systems [17]. In contrast, this survey is explicitly centered on AI agent systems developed for biological research. By organizing the literature around biological tasks and introducing a unified 5D taxonomy, we aim to systematically characterize how agentic designs align with biological data modalities, workflows, and evaluation constraints, offering a domain-specific synthesis grounded in current practice.

The remainder of this paper is organized as follows. The section titled “What is an Artificial Intelligence Agent in Biology?” clarifies the definition of AI agents in biological contexts and distinguishes them from foundation models. The section “Taxonomy of Artificial Intelligence Agents in Biology” introduces the proposed 5D taxonomy and reviews existing work within this framework. Finally, the “Discussion” section outlines key challenges, emerging domains, and future research directions.

## What is an artificial intelligence agent in biology?

### Defining biological agent

Generally, an AI agent in AI research is characterized as an autonomous computational entity that perceives and interprets the environment, formulates decisions, and selects actions through internal reasoning, and iteratively engages in self-improvement based on the feedback and observed outcomes. Unlike traditional static predictive models that passively map inputs to outputs, AI agents actively plan, act, and self-evaluate in dynamic environments, thus exhibiting the capacity for autonomous decision-making and adaptive behavior, which aligns closely with the pressing needs of contemporary biological research. Most biological problems cannot be solved via one-shot computational inference. Instead, they require iterative cycles across question formulation, literature sourcing, data processing, inference, and modeling, hypothesis evaluation and validation. In this case, AI agents are well suited to the dynamic, interactive, and co-evolving characteristics of biological research workflows.

Importantly, we emphasize that not all LLM-based systems or tool-augmented pipelines should be regarded as AI agents. Systems that merely execute single-turn inference, prompt-based reasoning, or one-shot RAG, without persistent internal state, autonomous task decomposition, or feedback-driven adaptation, remain fundamentally non-agentic. While such systems may exhibit strong representational or retrieval capabilities, they lack the process-level autonomy required to iteratively plan, act, evaluate, and revise analytical workflows. In biological research settings, where analytical objectives often evolve in response to intermediate results and uncertainty, this distinction is critical for separating agentic systems from static or semi-automated model pipelines. For example, language model-based frameworks such as GenePT [18] and scGPT [19], while effective for knowledge grounding and embedding-based representation learning, operate primarily through predefined prompting, and retrieval mechanisms and do not maintain an explicit planning loop, long-term memory, or self-directed decision-making. As a result, they should be viewed as powerful model-centric tools rather than full-fledged AI agents.

In biological research, an AI agent can be defined as an autonomy-enabled computational actor embedded within biological research workflows, capable of iterative goal-directed reasoning and decision-making. Such systems break down complex problems into actionable stepwise modules while dynamically adjusting their reasoning strategy in response to intermediate outcomes and uncertainty signals during analysis. They can further leverage external toolkits and related knowledge bases (e.g. CellChat, SCENIC,and LIGER) and, when necessary, interact with other computational systems or domain human experts to ensure research reliability and biological interpretability. Put differently, their value is process-oriented rather than output-oriented, leading to a traceable, reusable, and self-improving scientific pathway.

**Figure 1:**
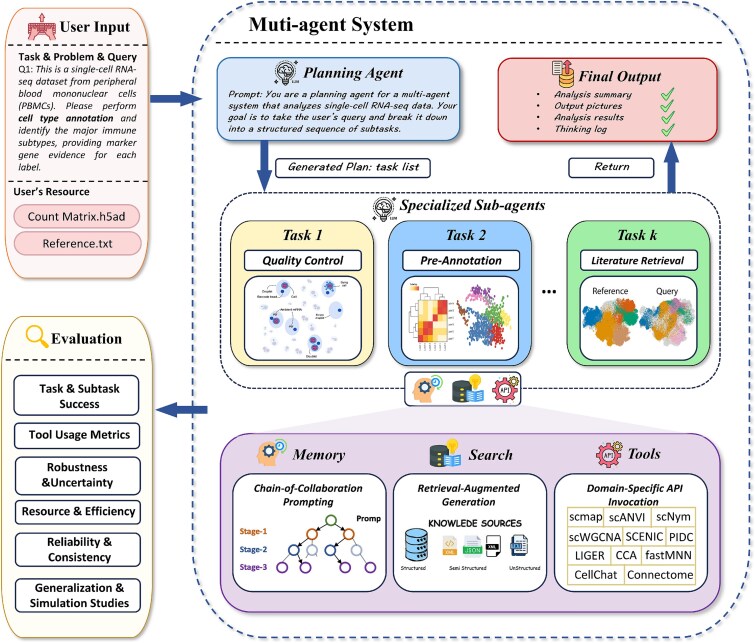
General workflow of a biological AI agent in which a Planning Agent decomposes user queries and biological resources into subtasks executed by domain-specific subagents that leverage memory, search, and tool invocation, with an evaluation module assessing robustness and reliability in a closed-loop analytical framework.

As illustrated in Fig. 1, a biological AI agent operates through a task–reasoning–action–feedback loop, enabled by a central coordinator that integrates multiple functional components. Upon receiving a biological query, the planning agent interprets the task and decomposes it into executable analytical steps, such as quality control, cell annotation, or literature retrieval. Subagents perform their specialized roles and share intermediate results via a shared-memory mechanism to maintain global consistency and traceability of reasoning. Moreover, the system leverages RAG for external knowledge incorporation and dynamically couples inference actions with established toolkits (e.g. scVelo [20] and CellChat [21]), while an evaluation module assesses robustness, uncertainty, and reproducibility at each stage of the workflow and in the final outputs. Based on such an interactive process, AI agents could promote human–machine collaborative intelligence, allowing models to contribute to biological discovery with scientific reasoning practices.

### Artificial intelligence agent versus foundation model

This article aims to survey the progress on recent agent-based studies in biological research. To better understand their conceptual and methodological uniqueness, it is necessary to contrast them with foundation models which currently dominate biological modeling. Foundation models such as *scGPT* [19], *scFoundation* [22], *Geneformer* [23], and *GeneMamba* [24] establish a powerful and robust framework for modeling high-dimensional biological data. Through training on large single-cell and multi-omics corpora, they learn generalizable molecular and cellular features that can be applied to diverse downstream analytical tasks, including cell type classification, trajectory inference, and gene expression prediction. These models typically follow a one-time inference paradigm, where outputs (e.g. embeddings or response features) are generated through a single forward computation. While this design already achieves strong performance and generality, it is intrinsically static, as inference depends on prelearned representations and lacks iterative reasoning, external tool coordination or dynamic decision planning, thus cannot actively adapt to dynamic research conditions or shifting user intents.

**Figure 2:**
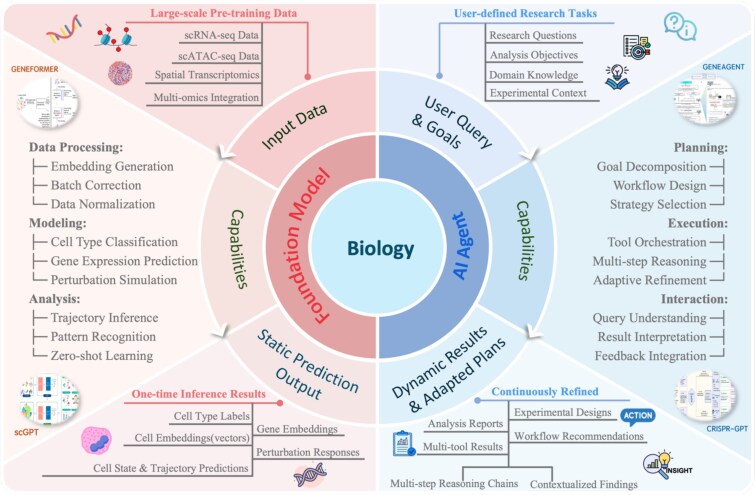
Conceptual comparison between foundation models and AI agents in biology. Foundation models focus on static prediction from large-scale pretraining, while AI agents enable dynamic reasoning, workflow orchestration, and adaptive refinement toward user-defined goals.

In contrast, AI agents aim to move beyond static inference toward dynamic reasoning and autonomous experimental workflows. As depicted in Fig. 2, agents commonly adopt a foundation model as their core reasoning engine but further extend them by integrating additional modules for planning, action, and interactive coordination. This design enables AI agents to interpret scientific queries, partition them into stepwise operations, select suitable tools or resources, inspect intermediate results, and update the workflow iteratively. These processes endow agents with the capacity of contextual sensitivity and adaptivity that transforms them beyond passive predictors to co-investigators in the scientific discovery process. We show a high-level comparison between AI agents and foundation models in biology in Table 1.

**Table 1:** Comparing AI agents with foundation models in biology.

**Capability**	**Foundation models**	**AI agents**
Representation learning	✓	$\times$
Static prediction	✓	$\times$
Multi-step planning	$\times$	✓
Context-aware reasoning	✓	✓
Tool usage and orchestration	$\times$	✓
Feedback-based adaptation	$\times$	✓

## Taxonomy of artificial intelligence agents in biology

**Figure 3:**
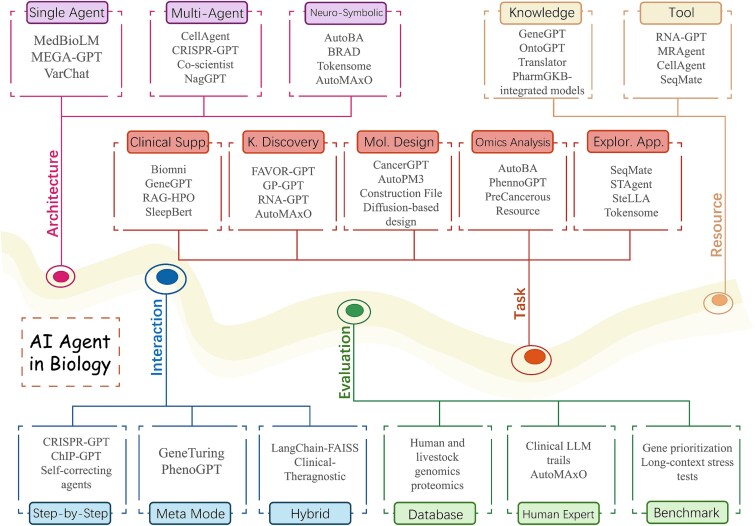
Taxonomy of AI agents in biology organized across five dimensions: architecture, task, interaction, resource, and evaluation, highlighting complementary functional perspectives on how agentic intelligence operates and evolves within biological systems.

To illustrate the progress of research on biological intelligent agents more systematically and clearly, we introduce a unified taxonomy consisting of 5D: **Biological Tasks**, **System Architectures**, **Evaluation Strategies**, **Interaction Modes**, and **Resource Integration**. Each dimension corresponds to a different function of research, and together they describe how biological AI agents function in real-world scientific environments and how they contribute to biological research. The taxonomy is shown in Fig. 3.

The proposed 5D taxonomy is intended as a structured abstraction of functionally orthogonal aspects of agentic intelligence in biological research. Specifically, each dimension answers a distinct and non-overlapping question: *what* biological problem the agent addresses (**task**), *how* cognition and coordination are organized (**architecture**), *how* reasoning unfolds over time (**interaction**), *how* success, robustness, and reliability are assessed (**evaluation**), and *how* external knowledge, tools, and data resources are incorporated (**resource**). Removing any single dimension would collapse important conceptual distinctions—e.g. architectural design alone cannot explain differences in interaction dynamics or evaluation protocols—while jointly these 5D are sufficient to uniquely characterize all surveyed agentic systems.

We further clarify the boundaries of this taxonomy through borderline cases. For instance, foundation models such as *scGPT* or *Geneformer* exhibit strong representation learning capabilities but lack autonomous planning and iterative interaction, and thus occupy only a limited subset of the taxonomy. Similarly, tool-augmented LLM systems such as GeneGPT demonstrate effective resource integration but remain non-agentic without persistent memory or closed-loop reasoning. These examples illustrate how the taxonomy distinguishes fully agentic systems from static or partially automated pipelines, while maintaining conceptual consistency across diverse biological applications.

In the following sections, we will expand the discussion of each subfield to examine how agents manifest themselves in various biological contexts and problem types.

### Biological tasks

Current research on biological AI agents spans several major task domains. Clinical diagnosis and treatment are the most studied areas, where agents typically use reasoning to analyze medical texts and patient records to assist in diagnosis, triage, or the development of personalized treatment plans. Knowledge discovery agents mine knowledge from biomedical literature and omics data to generate mechanistic hypotheses or gene–disease associations. Molecular design agents utilize generative optimization algorithms to generate new compounds and therapeutic proteins. Omics analysis agents manage large-scale transcriptomics, genomics, and spatial omics data, combining generative reasoning with retrieval-based or simulation-based optimization.

Across these domains, biological tasks differ primarily in data modality, workflow structure, and decision constraints that together shape how AI agents are integrated and what forms of value they provide. Rather than replacing task-specific analytical models, AI agents typically operate at the workflow level: they interpret high-level objectives, decompose tasks into executable steps, coordinate domain tools or models, and iteratively assess intermediate outcomes. This agentic role enables enhanced scalability, contextual reasoning, and process traceability, while task-specific limitations, such as regulatory oversight in clinical settings or experimental feedback scarcity in molecular design, continue to constrain the degree of autonomy achievable in practice.

#### Clinical decision support

Recent studies have shown that agents can help in clinical decision-making, but their role is limited. In oncology, agents’ recommendations can be innovative at times but can also be vague; e.g. these suggestions are often complementary rather than alternatives [25]. Optimized architectures, such as SensitiveCancerGPT and CART-GPT, have achieved significant improvements in drug sensitivity prediction and mapping T-cell status to treatment response, indicating the high potential of domain-aware agents [26, 27]. In gynecologic oncology, iteratively prompted GPT-4 is highly consistent with decisions of tumor committees, but tends to overtest and undertest surgical details, highlighting the importance of human supervision [28]. In addition, agents designed to improve physician–patient communication exhibit varying capabilities. The chatbot systems used to provide the results of the APOL1 test and information on the risk of hereditary cancer were rated well in terms of tone, but precision varied [29]. The testing for rare disease consultations showed that both GPT-4 and Gemini were highly reliable, but could still cause confusion at times, thus recommending their use as adjunctive tools rather than prescriptions [30, 31]. Real-world research indicates that patient acceptance depends more on previous healthcare experience than demographic characteristics, highlighting the importance of expanding coverage through accessible and trustworthy infrastructure [32].

In genomic analysis, biological AI agents demonstrate significantly superior recovery and tool expansion capabilities compared with the original LLM model. The RAG–HPO (human phenotype ontology) model significantly enhances the mapping ability from phenotype to HPO [33], while the GeneGPT model achieves a new breakthrough in the field of genome question answering using the NCBI API [34]. However, due to a bias towards known genes [35, 36], agents fail to outperform the screened process in phenotype-based gene prioritization tasks. In multimodal fusion, Biomni and BioLab combine reasoning, retrieval, and code execution in a variety of fields, from rare disease diagnosis to drug reuse [37, 38]. SleepBert demonstrates superior performance by combining polysomnography with literature retrieval [39]. However, other studies have also pointed out that biological AI agents lack a practical evidence base, gene classifiers contain confounding factors, and there is a potential risk of misuse for dual purposes [40, 41]. These papers argue that AI agents can only realize their maximum value when they are carefully constructed on domain knowledge, deployed under clinical supervision, and placed in easily accessible healthcare settings [42–44].

The strong representation of clinical applications primarily reflects differences in data modality, regulatory infrastructure, and task maturity rather than an inherent prioritization of clinical domains. In particular, clinical notes and electronic health records constitute large-scale, text-rich, and longitudinal data sources that naturally align with the language-based reasoning, memory, and planning mechanisms of contemporary AI agents. Such data are often standardized, continuously generated, and embedded within established decision-making workflows, making them more readily amenable to agent-based task decomposition, retrieval, and iterative validation than many experimental biology settings.

#### Knowledge discovery and hypothesis generation

In the field of knowledge discovery, biological AI agents can transform unstructured text and various omics data into machine-executable information with a clear source. For example, locally, an RAG-based proximal workflow compiles citation summaries and candidate relations from theliterature into queryable graphs through an iterative “scraping $\rightarrow$ structuring $\rightarrow$ hypothesis generation” loop [45–48]. Domain consoles can anchor collected entities to controlled vocabularies or clinical ontologies, thereby facilitating cross-cohort traceability [49–51]. Other researchers use bioinformatics management agents to map biological concepts and hypotheses to MAxO/HPO/MONDO databases, combined with human-participated audit trails, ensuring the hypothesis list is constantly growing and always auditable [52].

Regarding biological hypothesis generation, some studies use gene and cell-specific embeddings from literature or sequences to rapidly generate prior information. This information is then used as a reference for clustering, pathway analogies, and cell type associations [18]. Task-centric LLM further extends this process: FAVOR-GPT transforms annotations into conversational, evidence-relevant interpretations; GP-GPT aims to achieve gene-phenotype mapping; and RNA–GPT combines sequences with text to support query-conditional questions [53–55]. A lightweight embedding analogy-based approach reveals drug–gene relationships through vector geometry and pathway enrichment [56].

For evidence gathering, some domain-optimized encoders achieve high recall in protein–protein interaction mining, while some uncertainty-aware LLM adapters transform the extracted results into hypotheses with confidence scores for curators to explore [57, 58]. Dialogue analysis agents translate natural language queries into statistical manuals on a unified tumor database, modeling known associations, and proposing subgroup signals [59].

Biological agents can help scientific research carry out multiple verifications, improving scientific rigor. Studies have shown that research agents using these tools can formulate, critique, and modify designs for gene perturbation experiments that are traceable to the literature and outperform simple baseline models [60]. Agents can also act as “reward function searchers,” transforming fuzzy requirements into explicit reward codes for multi-objective optimization [61]. Furthermore, AI-driven Idiopathic pulmonary fibrosis (IPF) programs based on proteomic clocks can study processes from fibrosis to aging [62]; and generative pathogenicity models can transform sequence patterns into experimentally verifiable variant-mechanism associations [63].

#### Molecular and drug design

Molecular design and therapeutic research increasingly utilize biological AI agent methods. For example, diffusion-based platforms design synthetic DNA regulatory elements to regulate chromatin accessibility and gene expression in a cell type-specific manner [64]. Interoperable standard representations, such as build file specifications, encode molecular cloning workflows into modular objects for AI reasoning and simulation, facilitating the transition from computer design to the workbench [65].

Biological AI agents can also aid in drug discovery. CancerGPT translates the problem of predicting drug synergies into natural language reasoning, demonstrating excellence in rare cancer research with limited structured data [66]. The root node of AutoPM3 illustrates an LLM-based retrieval process that automatically collects evidence-based variant interpretation information for therapeutic target identification and precision medicine [67]. Besides, biological agents can enhance inorganic and chemical design. Evolutionary optimization driven by pretrained language models helps identify transition metal complexes with desired electronic properties and overcomes multi-objective optimization challenges in therapeutic applications, such as catalysis, imaging, and drug activation [68]. At the system-wide level, generative AI can supplement small datasets by generating realistic phenotypes, genotypes, and environmental scenarios, as discussed in predictive breeding research that can improve the accuracy of genome predictions [69]. Genome-wide association studies (GWAS) analysis shows that genetic association signals pave the way for the discovery of candidate molecular targets and can be applied to pharmacogenomics in human health [70].

#### Multi-omics analysis

In the field of multi-omics integration, biological agents help elucidate a variety of biological processes and disease mechanisms. For example, integrated analysis of proteomic quantitative trait loci, GWAS, and transcriptomics can help identify pathogenic genes for autoimmune diseases, such as candidate drug targets TNFRSF14 and CCL19 [71]. Multimodal mapping combining mass spectrometry and imaging techniques has revealed protein assembly and its association with cancer pathology [72]. Resources such as the PreCancerous Molecular Resource collect genomic, epigenomic, proteomic, and transcriptomic data across various cancer types to document molecular changes occurring in precancerous states [73]. Potential clinical applications include multi-omics strategies combining genomic, transcriptomic, proteomic, and lifestyle data structures for pharmacogenomics prediction and personalized treatment interventions [74]. Agents can also supplement medical education by generating examples of rare genetic syndromes (in the form of synthetic patients) and increasing phenotype–genotype learning datasets [75].

Furthermore, the use of biological agents expands the accessibility of multi-omics. Systems such as AutoBA simplify the design of omics workflows and the simpler parts of their execution [76]. Natural language models convert phenotypic descriptions in clinical texts into standardized datasets, thereby linking unstructured patient records with structured omics resources [77]. These findings suggest that biological agents are increasingly becoming key to enhancing the scale and diversity of omics data.

#### Exploratory applications

Exploratory and cross-disciplinary uses of AI agents bring together education, research, computational modeling, and policy. Language-based AI imitates biophysical insights in prediction of structural features of amino acids and viral protease–ligand complexes [78]. Transcriptomics pipelines, such as SeqMate, and STAgent are automating RNA-seq and spatial transcriptomic analysis including integrated interpretation, reducing the workflow from weeks to less than a minute [79, 80].

In the field of biomedical education, comparison studies demonstrate that generative AI models are superior to undergraduates in molecular biology examination [81]. Systems such as SteLLA employ RAG to mark student responses with detailed feedback [82]. GPT-based explainers for genetic programming allow users to interpret computational models [83].

On the translational side, case studies in rare diseases [84], chatbot evaluations for skeletal biology [85], and a phenotype recognition system such as PhenoBCBERT enhance mapping of clinical notes into standardized ontologies [86]. Foundation models in health survey their scope across omics, imaging and clinical language and discuss interpretability and fairness challenges [87]. Interestingly, regulatory research has also highlighted barriers in global collaboration for genomic AI development [88].

### System architectures

In this section, we focus on architectural design principles and execution mechanisms, rather than task-specific applications. Examples are used only to illustrate how different architectures operate in practice, not to evaluate task-level performance. The architecture of a biological AI agent not only determines the operational workflow but also fundamentally shapes its scalability and interpretability in complex biological contexts. Therefore, we use architecture as a key organizing framework in this review. We first examine two architectural paradigms in recent biological AI applications: single-agent systems that perform targeted tasks independently, and multi-agent systems that coordinate multiple specialized modules to manage more complex pipelines. We further observe a growing adoption of neuro-symbolic agents that motivates us to discuss them as a distinct architectural paradigm in this session.

#### Single-agent systems

Single-agent systems adopt a centralized execution model in which a single reasoning core sequentially performs task interpretation, tool invocation, and result validation. Their design prioritizes architectural simplicity, low coordination overhead, and tight coupling between reasoning and action within a unified control loop. Research on single-agent systems demonstrates that combining retrieval augmentation with domain-specific fine-tuning enables agents to achieve near-expert performance on well-defined biomedical tasks. Retrieval augmentation allows the model to access structured knowledge bases or curated literature, ensuring the reasoning process is traceable and supported by evidence, while domain-specific fine-tuning helps the model interpret, reason, and act in the logic, i.e. consistent with professional biomedical practice. By combining external knowledge grounding with internal reasoning alignment, the model improves from “general answering” to “answering with evidence like an expert.”

Retrieval-Augmented Reasoning. This addresses a central limitation of general models: they cannot directly access external information or provide evidence beyond the training corpus. For instance, VarChat shows how retrieval can integrate scattered literature on genomic variants, thereby speeding up clinical interpretation [89]. MEGA-GPT embeds retrieval into its workflow to support evolutionary genomics analysis [90], while MedBioLM boosts biomedical QA performance by integrating retrieval with fine-tuned reasoning [91]. These examples indicate that retrieval is not merely a memory aid but an important part of the reasoning process enabling agents to construct traceable evidence-based arguments.

Clinical Decision Support. In clinical practice, single-agent systems can achieve performance comparable to that of specialized clinical teams or software for tasks with well-defined decision criteria and abundant training data. In high-risk clinical settings, GPT-4-based single-agent systems have shown strong alignment with treatment decisions made by multidisciplinary breast cancer tumor boards [92]. For rare disease gene prioritization, professional-tool-level equivalence could be achieved through refined prompt engineering strategies [93]. However, such performance depends strongly on how the task is structured. When clinical decision-making requires integrating diverse expertise (e.g. oncology, pathology, genetics, and patient preference), a single-agent model may fail to reproduce the nuanced trade-offs made by a multidisciplinary team.

Computational Problem Solving. Beyond clinical workflows, single-agent systems can also function as autonomous solvers for specialized computational biology tasks. For mechanical design, iteratively generating solutions using finite-element analysis feedback converges more quickly than standard optimization techniques [94]. In omics analysis, single-agent systems can generate functional code that closely mirrors expert-designed workflows used from benchmarks like the DREAM Challenge [95]. Infrastructure advances such as fully pipelined distributed Transformers extend feasible context lengths to the scale of whole genomes or multi-omics maps, while domain-specific frameworks have successfully detected biomarkers and inferred biological pathways from gene expression data [96].

In conclusion, single-agent systems achieve relatively strong performance at pattern recognition, literature synthesis, and generating code for well-defined analytical workflows, whose effectiveness is largely determined by how modular the task is and how much relevant knowledge can be retrieved. However, they struggle in multi-step experimental planning, error diagnosis across heterogeneous data modalities, and coordination of multiple reasoning modes (e.g. integrating statistical analysis, mechanistic explanation, and experimental validation), leading to the increasing adoption of multi-agent system designs.

#### Multi-agent systems

In contrast to single-agent architectures, multi-agent systems distribute reasoning, execution, and verification across multiple specialized agents that interact through explicit communication and coordination protocols. This architectural shift enables parallelism, fault tolerance, and modular extensibility, at the cost of increased system complexity and coordination overhead. Multi-agent systems overcome the scalability and robustness limitations of single agents by decomposing complex workflows into specialized roles that coordinate through structured communication protocols. This design adds two core strengths: role-based specialization and reliability gained from collaborative cross-checking. To clarify the design principles of multi-agent systems, we identify three core architectural characteristics as follows.

Hierarchical Task Decomposition. For example, CellAgent organizes single-cell and spatial transcriptomics analysis hierarchically, coordinating specialized agents to collaboratively decompose tasks, invoke domain-specific toolkits, and iteratively improve the results through self-reflection [10]. The hierarchical structure implicitly encodes the workflow: the preprocessing agent handles quality control, the analysis agent applies statistical analysis, and the interpretation agent synthesizes biological insights. This design boosts efficiency while still supporting natural language interaction, indicating that decomposition lowers cognitive load per agent while keeping the workflow coherent overall.

Central Coordination of Specialized Modules. Coscientist adopts a GPT-4 planner to coordinate specialized modules such as web search, document reading, code execution, and experimental APIs [97]. Each module contributes specialized routines, allowing the system to handle complex workflows and heterogeneous tasks, though this architecture requires carefully designed inter-module communication and task allocation strategies.

Error Correction and Oversight. A key advantage of multi-agent systems is that they can embed supervisory layers that monitor and correct errors. For example, NagGPT integrates supervisory modules as a safeguard against hallucinations and reasoning drift [98], while CRISPR-GPT coordinates reasoning, planning, and execution agents to support gene-editing workflows, including protocol drafting, sgRNA design, and troubleshooting [5]. By allocating tasks among coordinated modules, such systems can support longer workflows, combine diverse data sources, and introduce self-monitoring to reduce risk in pharmacogenomics and causal inference applications.

**Figure 4:**
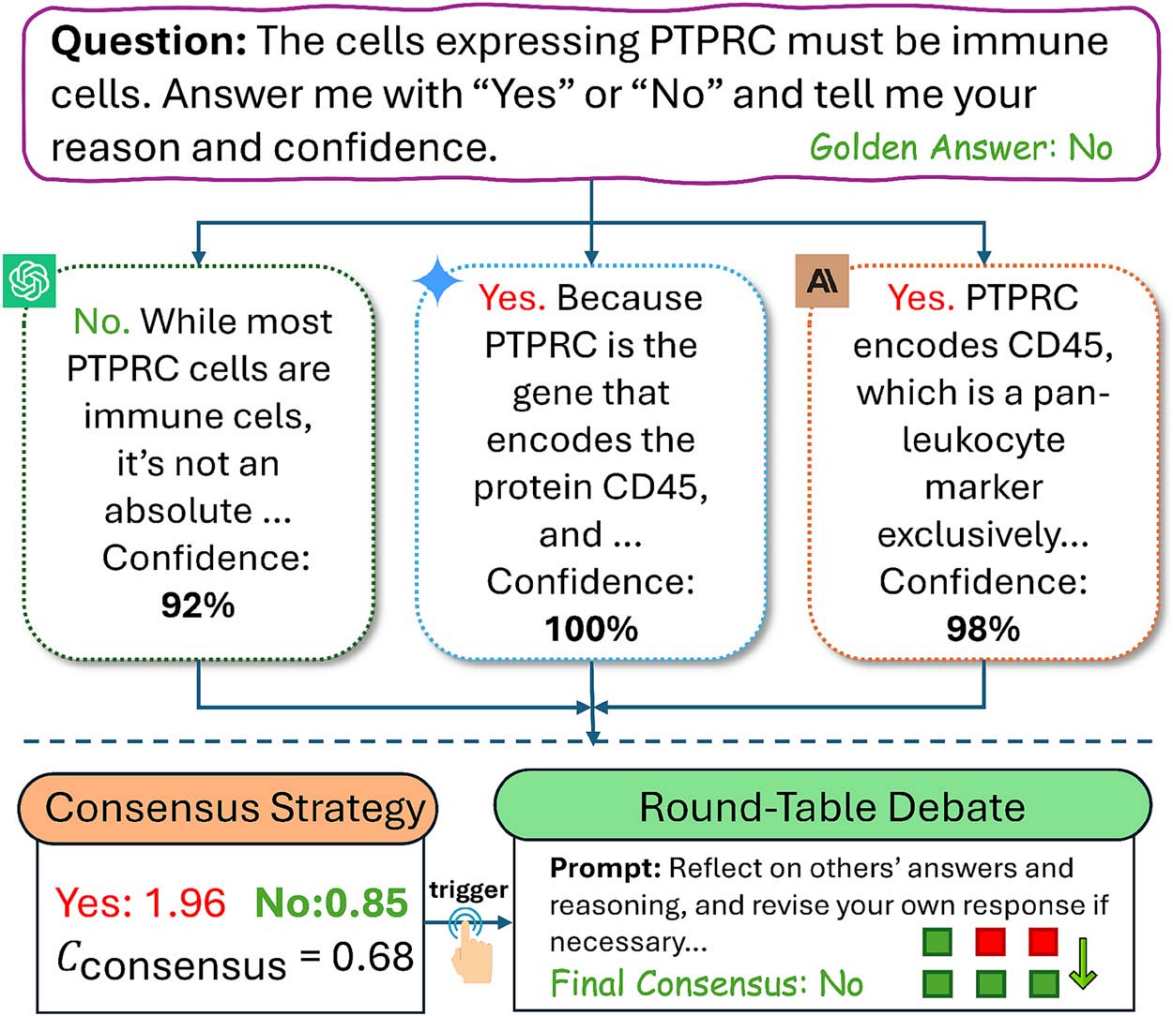
Multi-agent collaboration mitigates hallucination in biological reasoning by integrating divergent LLM responses to a PTPRC-based cell (protein tyrosine phosphatase receptor type C) identity query through confidence-weighted consensus and structured debate to produce a validated final decision.

In Fig. 4, we provide an illustration of the correction and oversight mechanism used in the multi-agent system to mitigate hallucination in biological reasoning tasks. In this example, three LLMs are independently queried with the question “The cells expressing PTPRC must be immune cells. Answer with ‘Yes’ or ‘No’ and provide the reasoning and confidence.” Although the ground truth is “No,” two models incorrectly answer “Yes” with high confidence by overgeneralizing the association between PTPRC (CD45) and immune cell identity. Only one model correctly notes that PTPRC expression is not strictly exclusive to immune cells. To resolve disagreement, a confidence-weighted consensus strategy is applied, followed by a round-table debate step, where models exchange and critique their reasoning. This collaborative refinement process shifts the final decision toward the correct answer (“No”), demonstrating that structured, multi-model collaboration can effectively reduce overconfident hallucinations and improve reliability in cell type identification and related biological inference tasks.

Overall, multi-agent systems significantly improve scalability and robustness in complex biomedical workflows by distributing roles and coordinating agent interactions, enabling the system to maintain both flexibility and domain-specific depth across diverse biomedical tasks. However, this architectural complexity introduces additional design and maintenance costs. The coordination protocol among agents must be carefully designed to balance the information sharing against communication overhead. Likewise, task assignment strategies must balance the gains from specialization against the risk that over-decomposition fragments reasoning. Furthermore, system debugging becomes more challenging, as errors may originate from individual agents, communication pathways, or emergent interaction dynamics. Despite these challenges, multi-agent architectures are becoming indispensable when both breadth and deep domain reasoning are required.

#### Neuro-symbolic integration

Neuro-symbolic integration represents a strategy applicable to both single- and multi-agent systems, in which explicit symbolic structures are used to guide and constrain neural computation. Unlike purely neural architectures that learn implicit patterns or traditional symbolic systems that require extensive manual encoding, neuro-symbolic agents couple data-driven perception with structured knowledge representation. This integration operates across multiple layers: the perceptual grounding layer, where neural models provide multimodal representations; the cognitive control layer, where symbolic ontologies ensure factual grounding and interpretable decision pathways; and the procedural execution layer, where planning and verification translate symbolic intent into controlled actions.

Perception–Symbol Integration. In this line of work, Tokensome demonstrates how symbolic decomposition enhances neural reasoning in structured visual domains by tokenizing chromosomes into symbolic units and applying vision–language backbones to perform compositional reasoning over cellular genetic structures [99]. This approach highlights a key principle: when input data possess inherent compositional structure (e.g. chromosomes consist of bands and proteins consist of domains), explicitly representing such structure symbolically can guide neural attention and improve generalization.

Ontology-Constrained Generation. Agents augmented with biomedical knowledge can regulate the outputs of LLMs to ensure terminological consistency and logical coherence. For example, AutoMAxO combines PubTator extraction and OntoGPT templated generation to produce assertions aligned with the MAxO/HPO/MONDO ontologies, transforming free text into structured symbolic knowledge [52]. Similarly, clinical genomic agents built on resources such as Orphanet and GeneReviews support age-specific and context-aware inference by constraining generation to curated disease–gene associations [100]. These systems illustrate that ontology grounding is not merely a posthoc validation step, but an active constraint shaping the generative process itself.

Programmatic Reasoning Workflows. In the Mergen framework, analytical reasoning is operationalized as a symbolic workflow mapping natural language tasks into explicit stages, such as reading, curation, modeling, and reporting, iteratively refining outputs through error-driven symbolic feedback [101]. Precision-medicine graph validation pipelines similarly constrain neural proposals by cross-checking pharmacogenomic claims against curated biological networks [74]. By externalizing reasoning steps into symbolic programs, these systems gain transparency (each step is inspectable) and modularity (components can be independently verified or replaced).

Across these implementations, neuro-symbolic integration provides three central capabilities: interfaces for translating between neural and symbolic representations, knowledge grounding to maintain factual fidelity, and structured reflection mechanisms that enable accountability and interpretability. Emerging patterns show how symbolic knowledge and data-driven learning can be jointly orchestrated to produce transparent and reliable biological reasoning. Despite these advances, fundamental questions remain regarding optimal integration strategies: How much structure should be imposed *a priori* versus learned from data? When do symbolic constraints enhance neural reasoning, and when do they limit exploratory capacity? How should ontologies evolve to incorporate new biological knowledge without architectural redesign? We argue that these questions will shape the next generation of biological AI agents.

### Evaluation strategies

The evaluation of biological AI agents necessitates a conceptual departure from classical bioinformatics benchmarking. While standard metrics such as accuracy, AUROC, and sensitivity remain indispensable for assessing individual predictive components, they provide limited insight into the *agentic* qualities that emerge over extended interactions. In practice, a model may achieve strong predictive performance yet still fail as an autonomous analytical operator if it lacks coherent multi-step planning, robust mechanisms for error recovery, or the ability to respect the statistical assumptions underlying the tools it invokes.

To render these agent-specific properties operational and reproducible, evaluation must extend beyond static outputs to explicitly assess *process quality* and *operational reliability*. These agent-specific evaluation dimensions are summarized in Table 2. Agent performance can then be characterized along several complementary dimensions. First, end-to-end task success under explicit constraints reflects whether an agent can complete a full analytical workflow within bounded steps, time, and tool budgets while adhering to prescribed protocols. Second, planning and re-planning correctness captures the alignment between intermediate steps and overall objectives, as well as the agent’s capacity to revise its strategy following interruptions or detected inconsistencies. Third, tool-use reliability concerns not only selecting appropriate computational or database tools, but also invoking them correctly, avoiding parameterization errors, output misinterpretation, or violations of underlying statistical assumptions. Fourth, evidence grounding and provenance assess the extent to which claims are supported by verifiable database identifiers or primary literature, thereby limiting unsupported or hallucinated assertions. Fifth, error management and recovery assess whether an agent can identify realistic biological or computational failure modes, such as species mismatches, gene symbol normalization issues, batch effects, doublets, or tool failures, and whether appropriate corrective actions lead to improved downstream outcomes within a reasonable number of steps. Sixth, memory consistency and state tracking measure whether critical intermediate artifacts, including gene lists, cluster assignments, and parameter settings, remain coherent and faithful across analytical stages. Finally, long-horizon robustness examines the stability of agent behavior under benign perturbations and its resistance to drift during prolonged interactions.

**Table 2:** Agent-specific evaluation dimensions beyond classical bioinformatics benchmarks.

**Dimension**	**Description**
End-to-end task success	Completion of full analytical workflows under explicit step, time, and tool constraints.
Planning and re-planning	Alignment between intermediate steps and overall objectives, including strategy revision.
Tool-use reliability	Correct selection and invocation of tools, including valid parameterization and assumptions.
Evidence grounding	Support of claims by verifiable databases or primary literature.
Error management	Detection and recovery from realistic biological or computational failures.
Memory consistency	Coherent tracking of intermediate artifacts across analytical stages.
Long-horizon robustness	Stability of agent behavior under perturbations and prolonged interactions.

Collectively, these dimensions capture aspects of agent behavior that classical benchmarks are not designed to measure. Rather than focusing solely on the accuracy of a final answer, they assess whether an agent can function as a reliable biomedical operator, including its ability to justify intermediate decisions, recover from errors, and maintain reproducibility across complex, multi-step, tool-mediated workflows.

#### Automated model-centric evaluation

Recent biomedical benchmarking efforts increasingly emphasize reproducibility, multi-scale data integration, and cross-domain comparability. For example, in human genetics, causal inference frameworks built on large-scale pQTL and disease cohort data have established reproducible evaluation standards for shared genetic architecture, effect directionality, and effect size [71]. In gene prioritization tasks, biological agent systems reveal how literature frequency bias and positional bias influence prioritization outcomes by varying parameters such as candidate gene set size, phenotype granularity, and task input ordering [93]. Furthermore, in animal genomics, shared pedigree, genotypic, and phenotypic information resources help establish reliable benchmarks for predictive models in the presence of real-world heterogeneity [102]. Systems such as GOLF, trained on AlphaFold structures, can perform uncertainty-calibrated pathogenicity predictions [63]. In livestock and ecological datasets, the authors examine whether agents can sustain their predictive power despite changes in the environment or over time [70, 103].

The evaluation methodology emphasizes reproducibility and transparency as well. Common practices when using agents emphasize the importance of verification, documentation, and standard workflows [104]. In the context of reinforcement learning, the design of the white-box reward function exemplifies how transparent methods can replace black-box optimization and produce more intuitive insights about agent behavior [61]. Benchmarks are increasingly requiring open code, versioned data, and standardized evaluation scripts to achieve replicability and ensure transparent comparison between laboratories [105].

Evaluation criteria change as AI agents become self-driven analytical systems, extending beyond mere performance to encompass operational reliability with human users and the quality of communication [95, 106]. These efforts map major evaluation categories across various domains: causal triangulation in human cohorts; multi-trait genomic pipelines in livestock; and mechanistic inference from interventional assays. Evaluation metrics are correspondingly diversified, including AUROC or RMSE for predictive accuracy, FDR-controlled discovery rates for causal inference, and calibration or interpretability assessments for mechanistic reasoning.

#### Human-in-the-loop and operational reliability

As biological AI agents evolve towards greater complexity, collaborative human–machine testing has become an increasingly important strategy for evaluating the reliability, safety, and domain consistency of biomedical AI applications. This approach directly integrates human expertise into the evaluation process, calibrates results, and ensures adherence to rigorous ethical and interpretative standards. Human experts are no longer passive annotators; instead, they are co-designers who define task frameworks, establish evaluation criteria, and interpret outputs within real-world clinical, educational, or research workflows.

In existing research in molecular oncology, GPT-based systems are integrated into clinically led oncology committee workflows to evaluate treatment options. Studies have shown that recommendations combining multiple models, based on consistency and evidence, outperform single models regarding clinical memorability and usability [25]. In genetic counseling, an expert panel evaluates the base model using standardized scoring criteria that emphasize professionalism, factual precision, and the impact on patients [30].

Such human-in-the-loop evaluations also apply to biomedical knowledge engineering and education. In ontology curation systems like AutoMAxO, AI agents perform high-recall extraction, while domain experts validate, correct, and enhance annotations through interactive interfaces. This cooperative pipeline substantially accelerates curation while maintaining medical accuracy [52]. In the digital health domain, the human-in-the-loop concept applies even at the system level. Experiments such as BRIDGE frame AI systems as interpreters of signals regarding patient engagement, while human administrators analyze behavioral data to develop equitable outreach interventions. These results emphasize the need to contextualize algorithmic predictions with human insight to ensure they generalize well, are fair, and respond to the realities of local health systems [32]. This iterative model locates the entire clinic, rather than just the clinicians or counselors within it, serving as evaluation anchors through judgment and domain ethics. In molecular biology examinations, researchers use educational benchmarks to compare the responses from agents and undergraduate students to official examination rubrics. The result suggests that, although agents can perform at or above human averages, expert judgment is required to ensure that surface fluency does not conceal conceptual or ethical mistakes [81].

From a methodological perspective, human-in-the-loop evaluation extends beyond numerical performance to multi-dimensional criteria. Common safeguards include reference verification, adjudications on mismatches, and consensus strategies that stabilize outputs by aggregating information from various models or raters. These mechanisms transform evaluation into a participatory governance process, ensuring that biological AI agents are robust, transparent, and reliable.

### Interaction modes

The interaction mode refers to how biological agents reason and communicate. Some agents use a step-by-step reasoning pattern with self-correcting loops to ensure transparent and iterative decision-making. Others adopt the zero-shot planner approach, also known as meta mode, that can automatically generate execution plans through a one-time structured prompt without the need for predefined workflows. There is also a hybrid mode, which integrates these two paradigms, making collaboration between human experts and automatic reasoning components possible.

#### Step-by-step interaction

In this mode, each step follows a fixed operational pattern: goal decomposition, planning, tool-based execution, and verification feedback. This structured approach creates an interpretable workflow that enables external inspection and verification of the internal reasoning process. Such transparency and reliability are highly valued in biomedical AI. For instance, CRISPR-GPT [5] exemplifies this approach through a multi-agent system where a Planner Agent decomposes goals into detailed task graphs, a Task Executor applies domain-specific tools with verification mechanisms, and a User-Proxy Agent maintains two-way communication with users throughout the process. ChIP-GPT [107] demonstrates another step-by-step interaction method, featuring a two-stage modular pipeline with a summary module that condenses database records into evidence chains and a question-and-answer module that assesses these records through controlled inference loops. In clinical decision support, some researchers propose distributing rationality across multiple specialized models. Advanced reasoning models structure problems into step-by-step subgoals, which are then translated into clinician-friendly natural language reports by other models. This pipeline architecture allows each model to focus on its specific strengths [108]. In program composition, interaction typically involves step-by-step dialogue where agents generate code based on user specifications, then automatically verify and execute it. Errors trigger a build–debug–repeat loop where the model diagnoses issues and refines the code iteratively. These error signals serve as valuable feedback for improved reasoning, though excessive complexity can impair convergence [101].

#### Zero-shot planner

Unlike step-by-step interaction that requires a predefined workflow, the zero-shot planner can dynamically generate execution plans by interacting with the language model using structured prompts. This paradigm shifts the focus from the management of multi-agent systems to the design of prompt model interfaces, where task constraints and operation modes are directly embedded in the model input. In zero-shot planners, a model directly includes an API pattern or database structure in the prompt to enable deterministic planning. In detail, the model acts as a program synthesizer, translating natural language intents into executable query sequences. It uses the biomedical information retrieval system and Web API to decompose complex genomic queries into executable API calls, obtaining precise results. The architecture of GeneTuring indicates that this pattern-based design can achieve state-of-the-art accuracy without fine-tuning, outperforming the retrieval enhancement model [34]. Another supplementary mode restricts the reasoning process to a structured vocabulary, thereby limiting the output space. In the phenotypic-driven gene prioritization task, the zero-shot planner suggested by human phenotype ontology (HPO) terms performed better than the model described by free text, reducing hallucinations and improving accuracy [35]. In tasks such as mathematics, chemistry, and biology, prompts constrained by LaTeX format can force symbolic reasoning, thereby achieving deterministic output [109]. Some prompts guide the generalization ability of the model through example-based templates. In scenarios where data are scarce, converting tabular data into structured text templates enable LLMs to achieve performance comparable to bigger models while maintaining zero-probability adaptability [66]. PhenoGPT further aligns the pretrained representation with the phenotypic annotation task through effective parameter tuning [86]. In biomedicine, this paradigm of zero-shot planners views language models as independent agents that can plan and execute, which reason dynamically through structured input constraints. It is suitable for knowledge-rich, yet data-scarce scenarios that need interpretability, reproducibility, and rapid hypothesis exploration without a step-by-step workflow. However, reliability remains a key challenge. The assessment of the genetic counseling task indicates that although GPT-4 is more accurate than GPT-3.5, it still generates outdated advice and inconsistent risk assessment [36].

#### Hybrid strategy

The above step-by-step approaches and zero-shot planners have achieved quite good results. However, the combination of algorithmic reasoning and human judgment is essential in high-risk biomedical applications. In the context of prostate cancer drug repositioning, researchers first use GPT-4 to give weakly supervised labeling. After that, humans correct uncertain labels and use these labels to train downstream classifiers. This hybrid approach has achieved better results than both fully automatic and manual systems, demonstrating that step-by-step approaches can effectively combine computational efficiency and expert adjudication [110]. Hybrid strategies merge the precision of step-by-step agents with the flexibility of zero-shot planners, which can balance automation with oversight. These systems create continuous feedback loops where retrieval, reasoning, and reflection work together under human supervision, allowing them to handle uncertainty while remaining interpretable. In this case, agents are not fully autonomous decision-makers but collaborative tools that are limited by built-in safeguards and clear validation processes. Some researchers use GPT-4 in LangChain-FAISS frameworks to automatically extract genetic traits, ensuring inferences align with databases and human review [47]. Clinical diagnostics and treatment demonstrate this paradigm, where AI–human systems assign initial pattern recognition to AI modules, then pass findings to domain experts who interpret results and make final treatment decisions [44]. These arrangements create adaptive task divisions: AI accelerates evidence synthesis and error detection, while humans provide contextual understanding and risk assessment. This partnership improves both efficiency and reliability by turning routine tasks into continuously learning processes shaped by feedback loops [111].

We argue that there are important tradeoffs in the agent architecture design across three aspects. First, the design of architecture determines how models balance autonomous ability with clinical oversight. Medical AI agents represent this tradeoff by achieving operational efficiency through increased autonomy while keeping safety through mandatory human supervision in high-stakes decisions. Moreover, data integration manages trade-offs between a wide range of retrieval and precise alignment, harmonizing different data types at the cost of increased computational complexity. Finally, we suggest that human–AI coordination balances efficiency against confidence. Faster automated decisions improve throughput but may reduce reliability, while extensive human oversight ensures accuracy but slows the process. Prompts and feedback protocols can be adjusted to match human reasoning patterns and risk tolerance.

### Resource integration

The dimension of resource integration concerns how agents use information from external sources. An increasing number of biological AI agents utilize domain-specific databases for genomics and proteomics, and these agents are connected with human experts for clinical validation. These additional sources of information and data enhance the model’s domain adaptation and significantly reduce hallucinations in large models.

#### Knowledge-augmented agents

In biomedical AI, a core design principle has emerged: models are no longer standalone predictors but coordinators that link unstructured data with structured knowledge resources. To ensure factual alignment and verifiability, systems are based on curated resources such as ontologies, knowledge graphs, and domain-specific databases. In detail, these authoritative repositories are used by models to retrieve, align, and serialize information. For example, in phenotype annotation, instruction-tuned models recognize mentions of phenotypes but frequently cannot correctly allocate the right HPO identifiers due to statistical representations lacking factual alignment. This reliability is recovered when external resources such as HPO API or semantic search are used to normalize label-to-ID mappings, which restrict the predictions inside curated term spaces [112]. Similarly, systems combining LLMs with resources like CPIC, PharmGKB, and ClinVar employ RAG, which is used to ground model inference in the curated knowledge of genes, drugs, and phenotypes [113–115]. For rare disease annotation, retrieval systems that extract from PubMed abstracts are used to fill OntoGPT with ontology-linked triples (grounded on MAxO, HPO, and MONDO); these are then verified by curators before becoming part of the graph infrastructures [52]. RAG models handle variant annotation in the same way, by including structured evidence, i.e. taken from genomics databases for explainable reasoning [116]. This follows best practice in pharmacogenomics where outputs are aligned with CPIC and PharmGKB guidelines, which keeps recommendations anchored in clinically validated resources [33].

Knowledge augmentation also expands to areas of social and ethical sensitivity. Knowledge-based clinical chatbots are used to integrate knowledge-based claims into culturally relevant clinical dialogues to promote understanding in underrepresented minorities [117]. Multilingual evaluation frameworks employ ontology-standardized resources that enable shared interpretation and responsibility among the languages and clinical contexts [118], thereby extending resource integration toward regulatory alignment with contained human oversight.

#### Tool-augmented agents

Tool-augmented agents connect professional bioinformatics tools to extend reasoning cores, functioning as controllers orchestrating complex workflows. Typically, this architecture comprises three components: knowledge access modules, computation toolkits, and governance components. Comprehensively, knowledge access modules can retrieve information from specialized databases, and computation toolkits are able to execute analytical functions. Moreover, governance components track actions, record provenance, and enforce quality control, which can transform reasoning into a stable computational procedure.

These design principles have been verified in practical applications. Multimodal RNA dialogue agents combine sequence encoders with language embedding models to achieve dual functions of molecular data retrieval and semantic interpretation [55]. The Mendelian Randomization Assistant integrates three key modules: ontology-based synonym expansion, GWAS data retrieval, and causal inference statistical analysis. These three modules can thereby transform the traditional analysis process into an iterative reasoning loop [119]. In the field of single-cell analysis, multi-agent systems adopt a hierarchical collaborative architecture, coordinating the three stages of planning, execution, and evaluation. This not only enables automatic algorithm selection but also retains a complete operational history record [10]. Furthermore, this design pattern demonstrates broad applicability across domains. The bioinformatics assistant connects the cognitive planner with the Text2SQL module and the analysis sandbox in order to transform user intentions into executable operation sequences while ensuring data privacy and result reproducibility at the same time [67, 79]. The infrastructure centered on retrieval combines topic modeling and vector search technology to support in-depth exploration of literature [120]. In addition, one agent can combine robot protocols with cloud laboratory APIs and independently plan as well as optimize experimental processes. It can also generate complete documentation records when executing the above actions for human review [97].

Overall, tool-enhanced agents can obtain structured resources through APIs, SQL queries, or laboratory protocols. By adopting a planning–acting–reflecting loop and maintaining traceable, type-safe tool calls, this architecture enables language models to coordinate knowledge acquisition, computational execution, and experimental operations with transparency and accountability.

### Summary

The representative systems summarized in Table 3 illustrate the growing diversity of AI agents designed for biological research. Each system reflects a distinct combination of architectural design, interaction pattern, evaluation method, and resource integration. Multi-agent architectures such as Biomni [37], CellAgent [10], and Co-Scientist [97] emphasize collaborative reasoning and distributed task decomposition, while neuro-symbolic models including AutoMAxO [52], Tokensome [99], and mergen [101] embed symbolic constraints or ontology structures within language-model pipelines. Tool-integrated systems such as GeneGPT [34], CRISPR-GPT [5], and NagGPT [98] connect LLMs to external APIs or experimental databases, enabling automated retrieval, validation, and execution of bioinformatics tasks. Step-by-step reasoning dominates in decision-support and protocol-generation agents, whereas zero-shot generalization is preferred in domain-adapted frameworks such as PhenoGPT [86], scWGBS-GPT [122], SleepBert [39], and RAG–HPO [33]. Evaluation strategies range from fully automated benchmarks to human-in-the-loop verification, reflecting different levels of experimental control in biomedical settings. Knowledge and tool integration remain central: systems such as BRAD [121] and AutoBA [76] link language models with curated biomedical ontologies and workflow tools to ensure traceability and reproducibility. Together, these systems demonstrate how recent AI-agent designs unify reasoning, tool use, and domain expertise to support diverse biological objectives—spanning clinical decision support, genomic query interpretation, molecular synthesis planning, and ontology-based curation—while revealing distinct design tradeoffs among architectural, interactive, and evaluative dimensions.

**Table 3:** Representative AI agent systems in biology and their comparative overview.

**System**	**Task domain**	**Architecture**		**Interaction**		**Evaluation**		**Resource**	
		Single	Multi	Step	Meta	Auto	Human	Knowl.	Tool
Biomni [37]	General biology tasks	–	✓	–	✓	–	✓	✓	✓
GeneGPT [34]	Genomic QA	✓	–	✓	✓	✓	–	✓	–
CellAgent [10]	Single-cell/spatial omics	–	✓	✓	–	✓	–	✓	–
GeneAgent [4]	Single-cell/spatial omics	–	✓	✓	–	✓	–	✓	–
CRISPR-GPT [5]	Gene editing	✓	✓	✓	✓	✓	✓	✓	–
BioLab [38]	Life science wet-lab	–	✓	–	✓	–	✓	✓	✓
AutoMAxO [52]	Ontology curation	✓	–	✓	–	✓	✓	✓	–
Tokensome [99]	Cytogenetic karyotyping	✓	–	✓	–	✓	–	–	–
Co-Scientist [97]	Molecular synthesis	✓	–	✓	–	✓	–	✓	✓
BRAD [121]	Biomarker discovery	✓	–	✓	–	✓	✓	✓	✓
AutoBA [76]	Multi-omics automation	✓	–	✓	–	✓	–	✓	✓
SteLLA [82]	Biomedical education	✓	–	✓	–	–	✓	–	–
PhenoGPT [86]	Pheno–geno mapping	✓	–	–	✓	✓	–	✓	–
scWGBS-GPT [122]	Methylation analysis	✓	–	–	✓	✓	–	✓	–
NagGPT [98]	Genomic query validation	✓	–	✓	–	✓	✓	✓	✓
ChIP-GPT [107]	ChIP–seq reasoning	✓	–	✓	–	–	–	✓	–
mergen [101]	Code synthesis for omics	✓	–	✓	–	✓	–	✓	✓
SleepBert [39]	Sleep data analysis	✓	–	–	✓	✓	–	✓	✓
RAG–HPO [33]	Pheno–ontology mapping	✓	–	–	✓	✓	–	✓	✓

## Discussion

**Figure 5:**
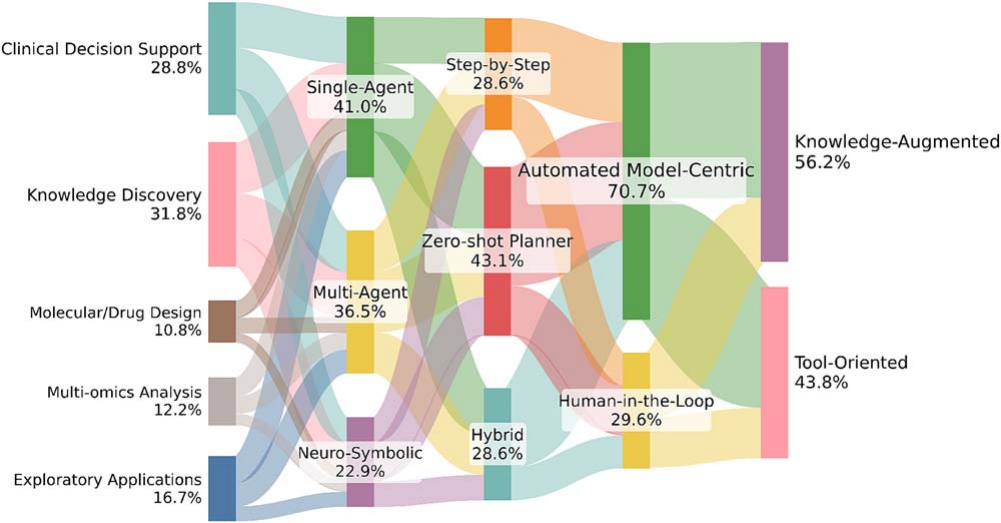
Cross-dimension relationships in our survey taxonomy across Tasks, Architectures, Interaction, Evaluation, and Resource Integration, with layer-normalized flow widths (summing to 100%) illustrating relative prevalence and typical transitions among surveyed systems. Minimal bridge flows ($\leq$0.2%) are included only to preserve visual continuity and do not indicate additional empirical association.

Based on the above review, current research on biological AI agents demonstrates both progress and limitations, and Fig. 5 summarizes the cross-dimensional relationships and coupling patterns observed across surveyed systems. Although these systems increasingly exhibit autonomy, interpretability, and domain adaptability, their practical application still faces conceptual and technical barriers. In the following section, we outline the major challenges and discuss some insights to guide future research. Accordingly, the following discussion focuses on identifying open challenges and articulating prospective research directions, rather than assessing near-term deployment readiness or predictive outcomes.

### Emerging and underexplored domains

Despite substantial progress in AI agents for biological research, existing systems remain unevenly distributed across biological domains. Highlighting these gaps is necessary to contextualize the scope of current agentic systems and to clarify where future methodological advances are most needed. Beyond the domains discussed above, emerging applications of biological AI agents are beginning to appear in areas such as plant biology, crop breeding, and ecological genomics. These domains remain underexplored due to limited standardized datasets and evaluation protocols, yet they offer promising opportunities for agentic systems to support hypothesis generation, phenotype–genotype association, and experimental planning at scale. Below, we summarize recent advances toward the application of AI agents in these less explored domains.

#### Closed-loop wet-lab experimentation

Recent perspectives articulate a vision of AI scientists that coordinate hypothesis formulation, experimental design, execution, and analysis through continuous feedback cycles [17]. In practice, however, most existing systems remain limited to protocol generation or downstream data analysis, with experimental execution and real-time adaptation largely controlled by human experts. Although early efforts in laboratory automation and self-driving laboratories demonstrate partial integration of AI with robotic platforms [123], no broadly applicable agent currently supports autonomous monitoring, failure diagnosis, and adaptive redesign across heterogeneous biological assays. Key technical barriers include hardware heterogeneity, noisy experimental outcomes, safety constraints, and limited grounding of agent reasoning in physical laboratory environments.

#### Ecological and evolutionary systems

Ecological and evolutionary systems are characterized by long temporal horizons, strong environmental coupling, and multiscale interactions that challenge standard data-driven workflows. While machine learning models have been applied to species distribution modeling, population dynamics, and environmental forecasting, such approaches typically operate as static predictors rather than adaptive agents [124]. To date, no comprehensive AI agent is capable of autonomously integrating heterogeneous ecological data sources, generating competing evolutionary hypotheses, and refining models through iterative simulation and observation. The scarcity of standardized datasets and the difficulty of validating long-term predictions further constrain the development of agentic systems in these settings.

#### Temporal and regulatory biological processes

A similar limitation is evident in domains requiring sustained temporal and regulatory reasoning, including developmental biology and clinical research. Recent agentic systems for spatial and single-cell analysis illustrate how autonomous reasoning can support complex biological workflows [125]; however, these systems primarily operate on snapshot data and do not capture long-range developmental trajectories or causal interventions. In clinical contexts, agentic frameworks have advanced decision support and workflow assistance [126, 127], yet comprehensive agents for clinical trial design and regulatory reasoning remain largely absent. Such applications require coordinated reasoning over ethical constraints, regulatory guidelines, patient heterogeneity, and evolving evidence, placing demands on agent autonomy that exceed those of current systems. As emphasized in perspectives on AI scientists, addressing these limitations is essential for extending agentic methods beyond analysis pipelines toward more general forms of scientific reasoning [17].

### Challenges

Although AI agents are transforming the field of computational biology, several fundamental technical, conceptual, and infrastructural barriers remain. Below, we outline six key challenges that define the current research frontier.

#### From chat-based assistants to end-to-end intelligent agents

At present, agents in the biomedical domain mostly remain text-based, directly invoking LLMs such as ChatGPT to act as question-answering systems that respond to user input through natural language generation. These systems perform well in conversational fluency and superficial reasoning but lack autonomy, planning, and action capabilities. They cannot decompose complex biological tasks into sequential subtasks, invoke computational tools, or adapt based on feedback. Therefore, they remain limited to a query–response mode rather than functioning as general-purpose intelligent agents capable of goal-driven analysis. In biology, many tasks such as single-cell RNA sequencing analysis, multi-omics integration, or CRISPR experiment design are not single-step problems, but require multi-stage analytical workflows involving coordination, iterative validation, and user feedback. A general-purpose biological agent should ideally move beyond question answering toward structured task interpretation, workflow planning, tool invocation, and intermediate evaluation; however, achieving such autonomy remains an open research challenge rather than a solved capability. Achieving such autonomy requires breakthroughs in multi-agent coordination, memory management, and task planning, which current chat-based systems cannot yet provide.

#### Reliability, hallucination, and factual grounding

Language models commonly produce “hallucinated” outputs, i.e. responses that appear plausible but are factually incorrect. In the biomedical domain, this problem can have serious consequences: an incorrect gene marker, pathway annotation, or diagnosis may mislead all subsequent analyses, and even put patients in a more dangerous condition. Although RAG can partially mitigate this issue, hallucination control remains unstable because most current systems lack multi-level verification or structured result evaluation. Furthermore, biological research involves integrating various forms of evidence, such as wet-lab experiments and literature, which cannot be fully obtained only through text description. Without explicit reliability safeguards, verification mechanisms, and uncertainty awareness, agent-generated analyses may introduce compounding errors, underscoring the need for cautious deployment and rigorous validation rather than unconditional automation.

#### Domain specialization and biological contextualization

General-purpose language agents are trained based on extensive text corpora, but they lack understanding of the complex characteristics of biological systems and are unable to directly interpret details such as cell type hierarchies, molecular interaction networks, or the nuances of experimental designs. For instance, a general agent might be able to recognize “T cells,” but it lacks contextual knowledge to distinguish between effector memory subsets and naive T-cell subsets, or to link differential expression results with previously validated lineage markers from previous studies. Similarly, in transcriptomics or proteomics, if an agent cannot access domain-specific knowledge bases, such as HPO, MAxO, or cell ontologies, it may result in incomplete or misleading conclusions. Effective biological agents, therefore, require domain grounding and contextual constraints that remain difficult to generalize across diverse biological subfields.

#### Data privacy, security, and ethical compliance

Biomedical and clinical data, including genomic data, transcriptomic data, and self-reported patient information, are all subject to strict privacy regulations such as HIPAA and GDPR. Cloud-based APIs used for model inference typically require the transmission of sensitive data to third-party servers that poses significant security risks. There is a fundamental contradiction here: the most powerful models are often closed-source and hosted in the cloud, while biomedical research requires local control, transparency, and data sovereignty. Resolving this tension will likely require regulatory, infrastructural, and architectural innovations, rather than purely algorithmic advances.

#### Standardized evaluation, reproducibility, and benchmarking

Although there is growing interest in biological AI agents, the field lacks a standardized evaluation framework. Most published systems are demonstrated through qualitative case studies or small-scale benchmarks, without repeatable metrics or open datasets. This fragmented evaluation landscape complicates objective comparison across systems and limits reproducibility. When proprietary APIs or closed-source models are involved, independent verification becomes particularly challenging, further emphasizing the need for transparent and standardized benchmarks.

#### Computational cost and scalability

Currently, most agents employ language models such as GPT-4, Claude, or Gemini as their reasoning backbone. While these models exhibit strong reasoning capabilities, they impose substantial computational and energy costs. Running long-context biological workflows or deploying agents on large-scale single-cell and multi-omics datasets often exceeds the capacity of typical academic or clinical infrastructure. As a result, scalability and sustainability remain practical constraints that may limit widespread adoption unless more efficient model designs and deployment strategies are developed.

### Insights

#### Agents as autonomous scientific collaborators

Next-generation biological AI agents are better viewed as prospective autonomous collaborators rather than mere language interfaces. Rather than serving only as information retrieval tools, they may assist in formulating objectives, executing workflows, and refining hypotheses based on iterative feedback. Through reasoning, memory, and tool usage, such systems could potentially simulate aspects of scientific practice, including experiment planning, evidence synthesis, and mechanistic hypothesis generation. This perspective reframes computation in biology as a process-oriented research assistant, although realizing this vision remains an open research agenda rather than an established paradigm.

#### Multi-agent collaboration as a form of robustness

At any scale of model size, there is no single solution that guarantees robust reasoning across all biological contexts. Multi-agent architectures, such as planners, executors, validators, and critics, offer structured redundancy and error-checking mechanisms. They may help mitigate hallucination and bias through consensus-building and uncertainty estimation. However, the effectiveness and scalability of such collaborative designs require systematic empirical validation across diverse biological tasks.

#### Lightweight, domain-agnostic models for fair access

Specialized small language models trained and fine-tuned for biomedical reasoning will contribute to enabling widespread analytical intelligence evolution. Through techniques such as distillation and continual learning, smaller models could preserve domain knowledge while operating within institutional computational constraints. Nevertheless, trade-offs between performance, generality, and efficiency remain to be carefully evaluated in real-world deployments.

#### Standardization and transparency as scientific infrastructure

With the increasing number of biological AI agents, standardized evaluation and transparent system design are becoming increasingly important. Open and modular architectures, uncertainty calibration, and reproducible workflows facilitate independent validation and reuse. Rather than a guaranteed outcome, such standardization should be viewed as a collective and incremental effort that evolves alongside the field.

Key Points

- The survey provides a timely and comprehensive synthesis of recent studies on biological artificial intelligence (AI) agents (e.g. Biomni and BioLab) across clinical analytics, molecular design, multi-omics computation, and knowledge discovery.
- The survey clarifies how biological AI agents differ from foundation models (e.g. scGPT and scFoundation) by enabling multi-step planning, contextual reasoning, workflow refinement, and tool-driven analysis.
- The survey proposes a unified 5D taxonomy, covering tasks, architectures, interaction modes, evaluation strategies, and resource integration, that organizes fragmented agent-based research into a coherent framework.
- The survey reviews representative single-agent, multi-agent, and neuro-symbolic systems, highlighting key design tradeoffs involving autonomy, interpretability, reliability, and biological grounding.
- The survey identifies major challenges—including hallucination control, privacy protection, reproducibility, and standardization—and outlines future directions for developing robust and transparent biological AI agent ecosystems.

## Data Availability

All materials discussed in the manuscript refer to previously published studies, datasets, and resources that are appropriately cited within the text. A collection of these paper is available at https://github.com/MineSelf2016/biological_agents_survey.
